# Functional force stimulation alters motor neuron discharge patterns

**DOI:** 10.3389/fnins.2023.1293017

**Published:** 2023-12-05

**Authors:** Yaodan Xu, Zuyu Du, Anyi Cheng, Runwei Lin, Kaichen Wang, Yibin Jin, Chenyun Dai, Lin Xu

**Affiliations:** ^1^School of Information Science and Technology, ShanghaiTech University, Shanghai, China; ^2^Shanghai Advanced Research Institute, Chinese Academy of Science, Shanghai, China; ^3^University of Chinese Academy of Science, Beijing, China; ^4^School of Information Science and Technology, Fudan University, Shanghai, China; ^5^Shanghai Engineering Research Center of Energy Efficient and Custom AI IC, Shanghai, China

**Keywords:** functional force stimulation, motor unit synchronization, high-density surface electromyography, blind source separation, discharge timings

## Abstract

**Introduction:**

Beneficial effects have been observed for mechanical vibration stimulation (MVS), which are mainly attributed to tonic vibration reflex (TVR). TVR is reported to elicit synchronized motor unit activation during locally applied vibration. Similar effects are also observed in a novel vibration system referred to as functional force stimulation (FFS). However, the manifestation of TVR in FFS is doubted due to the use of global electromyography (EMG) features in previous analysis. Our study aims to investigate the effects of FFS on motor unit discharge patterns of the human biceps brachii by analyzing the motor unit spike trains decoded from the high-density surface EMG.

**Methods:**

Eighteen healthy subjects volunteered in FFS training with different amplitudes and frequencies. One hundred and twenty-eight channel surface EMG was recorded from the biceps brachii and then decoded after motion-artifact removal. The discharge timings were extracted and the coherence between different motor unit spike trains was calculated to quantify synchronized activation.

**Results and discussion:**

Significant synchronization within the vibration cycle and/or its integer multiples is observed for all FFS trials, which increases with increased FFS amplitude. Our results reveal the basic physiological mechanism involved in FFS, providing a theoretical foundation for analyzing and introducing FFS into clinical rehabilitation programs.

## 1 Introduction

Skeletal muscle atrophy and disfunction are common neuromuscular diseases in elderly people and patients after long hospitalization or cancer treatment (Yin et al., 2018; Okun et al., 2021), leading to a large amount of movement disability and accidental mortality. Resistance training has been extensively employed for the recovery of skeletal muscles. It is able to enhance the neural drive in the first few training weeks, and then change the morphology, architecture, and size of the muscle tissue at a later stage (Sale, 1988; Karinkanta et al., 2010). In order to improve the ability of force generation, resistance training is expected to impose a moderate to high neuromuscular demand, e.g., >60 − 70% of 1 repetition maximum (Kraemer et al., 2002). This strategy is less suitable for elderly people or patients due to their reduced ability of fully activation of the muscles.

Mechanical vibration stimulation (MVS) has been reported to produce beneficial effects on muscle strength, power performance, bone density, and balance (Burke et al., 1970; Rubin et al., 2001; Cochrane and Stannard, 2005; Karinkanta et al., 2010). MVS can be directly applied to the muscle belly or tendon through a probe (Matthews, 1966; Godaux and Desmedt, 1975), or indirectly applied to the entire body through a vibrating platform such as the whole body vibration (WBV) platform (Cochrane and Stannard, 2005; Karinkanta et al., 2010). The observed beneficial effects during local or whole body MVS have been mainly attributed to a reflex contraction referred to as tonic vibration reflex (TVR) (Eklund and Hagbarth, 1966; Hagbarth and Eklund, 1966; Matthews, 1966). TVR originates from vibration-induced deformation in the primary spindle endings, resulting in reflex activation through the Ia-afferent and the spinal cord to the α-motoneuron. It is well documented that the outcomes of MVS depend strongly on the training parameters such as the amplitude and frequency of the vibratory stimulation as well as the initial contraction level of the muscle (Martin and Park, 1997; Karinkanta et al., 2010). These parameters are, unfortunately, not freely adjustable in the existing MVS systems, limiting their potential applications in fitness or clinical neuromuscular rehabilitation programs.

As an alternative, a novel vibration system has been proposed in recent decade (Xu et al., 2012). Different from MVS consisting of local or whole body mechanical displacement, vibration stimulation in the new system consists of sinusoidal force modulation generated by a motor, which is applied to the muscle via a dedicated bar-rope-handle interface (Xu et al., 2012). The training parameters of this system are fully adjustable through the software controlling the motor driver (Xu et al., 2012). In order to distinguish from the MVS, this force-modulated vibration is referred to as functional force stimulation (FFS) in the rest of this paper. FFS is reported to produce similar effects as MVS (Mischi and Cardinale, 2009; Xu et al., 2015a, 2016), which have also been, at least partially, ascribed to the spinal reflex, i.e., TVR.

However, the physiological mechanisms of response to TVR are in fact controversial. By the analysis of single motor unit (MU) action potential under local MVS, many studies have reported TVR to have a monosynaptic pathway, as evidenced by the time-looked (synchronized) MU discharges within the vibration cycle (Matthews, 1966; Homma et al., 1972; Godaux and Desmedt, 1975; Desmedt and Godaux, 1976). However, other studies have observed that, with local MVS applied to the human, some MUs are locked to the vibration cycle while others are not. These findings have been taken as indicators of the occurrence of both monosynaptic and polysynaptic activation of the motor neurons during TVR (Hirayama et al., 1974; Matthews, 1994).

Furthermore, most of the proposed mechanisms for TVR are based on the analysis of single MU action potential with locally applied MVS. The manifestation of TVR in WBV and FFS is in fact debatable. Some studies have associated the TVR-elicited MU synchronization during WBV or FFS with the sharp peaks at the vibration frequency and its harmonics in the amplitude spectrum of the surface electromyography (sEMG) (Martin and Park, 1997; Xu et al., 2015b). Other studies argue that these sEMG spectral peaks may be primarily due to motion artifacts generated by vibration-induced displacement in the electrode-skin interface (Abercromby et al., 2007; Fratini et al., 2009; Romano et al., 2018). These studies violate the manifestation of vibration-induced MU synchronization, and thus hamper our understanding of the physiological response of the muscles to WBV and FFS.

The debates in the manifestation of vibration-induced MU synchronization in WBV and FFS arise mainly from the indirect analysis of MU activation using global features extracted from sEMG, such as the root mean square value, mean frequency, and conduction velocity (Martin and Park, 1997; Abercromby et al., 2007; Fratini et al., 2009; Xu et al., 2015b; Romano et al., 2018). Needle EMG can directly measure the action potential of a single MU, but is unsuitable for the investigation of MU activation patterns during WBV and FFS training due to its invasive nature. Besides, needle EMG can only record local muscle activities within a limited area. High-density electrode grid enables the recording of multi-channel sEMG over the entire muscle, from which the underlying neural drive sent to the muscle by the motor neurons can be decoded using advanced decomposition algorithms (Holobar and Zazula, 2007; Chen and Zhou, 2015; Negro et al., 2016; Farina et al., 2017; Jiang et al., 2021).

The aim of the present study is therefore to investigate the effect of FFS on MU synchronization by examining directly the MU discharge timings decoded from high-density sEMG. Notch filtering at the vibration frequency and its harmonics is employed prior to sEMG decomposition irrespective of the nature of those spectral components being vibration-induced motion artifacts or muscle activity. Thus, the decoded MU discharge timings can provide reliable interpretation on the activation patterns of the motor neurons under different FFS conditions.

## 2 Methods

### 2.1 Subject

Eighteen healthy right-handed subjects (age = 28 ± 5 years, 12 males and six females) with no history of neurological diseases or injuries volunteered in this study. The experimental protocol was clearly explained to the participants before the experiment, and written informed consents were received from all subjects. This experiment was approved by the local Research Ethics Committee.

### 2.2 Experimental setup

#### 2.2.1 Functional force stimulation

**Figure 1:**
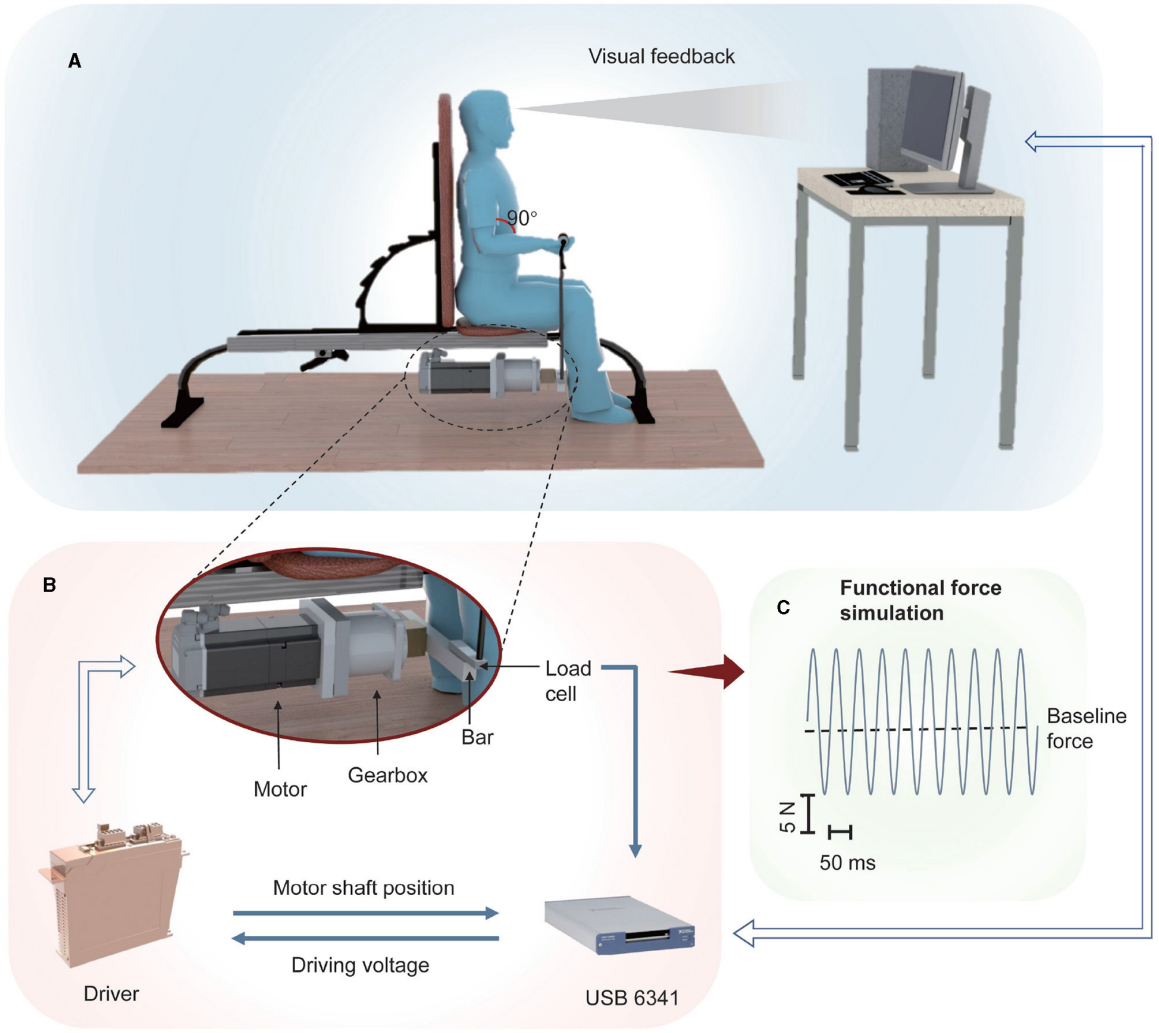
Scheme of the adopted FFS system: **(A)** A motor generates a sinusoidal force that is applied to the subject through a bar-rope- handle interface. A visual feedback of the position of the motor shaft, measured by a rotary encoder embedded in the motor, is provided to the subject through a LED screen; **(B)** The motor driver is controlled by a USB 6341 card, which is also employed to measure the encoder signal and the signal of a load-cell embedded in the bar aiming at detecting the generated force. **(C)** Example of the generated force.

FFS was generated by a dedicated vibration system realized in our previous study (Xu et al., 2012), as shown in Figure 1. A three-phase permanent motor (MSK060C, Bosch Rexroth, Boxtel, The Netherlands) was employed to generate FFS, which consisted of a baseline force with superimposed sinusoidal force modulation. The motor driver (IndraDrive HCS02, Bosch Rexroth, Boxtel, The Netherlands) was controlled through a USB 6341 card by a dedicated software implemented in LabView^Ⓡ^ (National Instruments, Austin, TX, USA). An aluminum bar was utilized to convert the rotary force to a vertical one, which was then applied to the muscle through the handle and rope connected to the bar.

The angular position of the motor shaft was measured by a rotary encoder embedded in the motor and the USB 6341 card connected to the PC. Real-time visual feedback reflecting the wrist position was therefore provided to the subjects through a LED screen in order to instruct them to perform the desired (isometric) contraction. Besides, a load-cell (LC62SP, OMEGA Engineering, Norwalk, CT, USA) was embedded in the bar, enabling real-time measurement of the generated force as well as the subject's maximum voluntary contraction (MVC). However, real-time force measurement was not performed in the present study but in a previous study for dedicated system calibration (Xu et al., 2012).

#### 2.2.2 EMG recording

**Figure 2:**
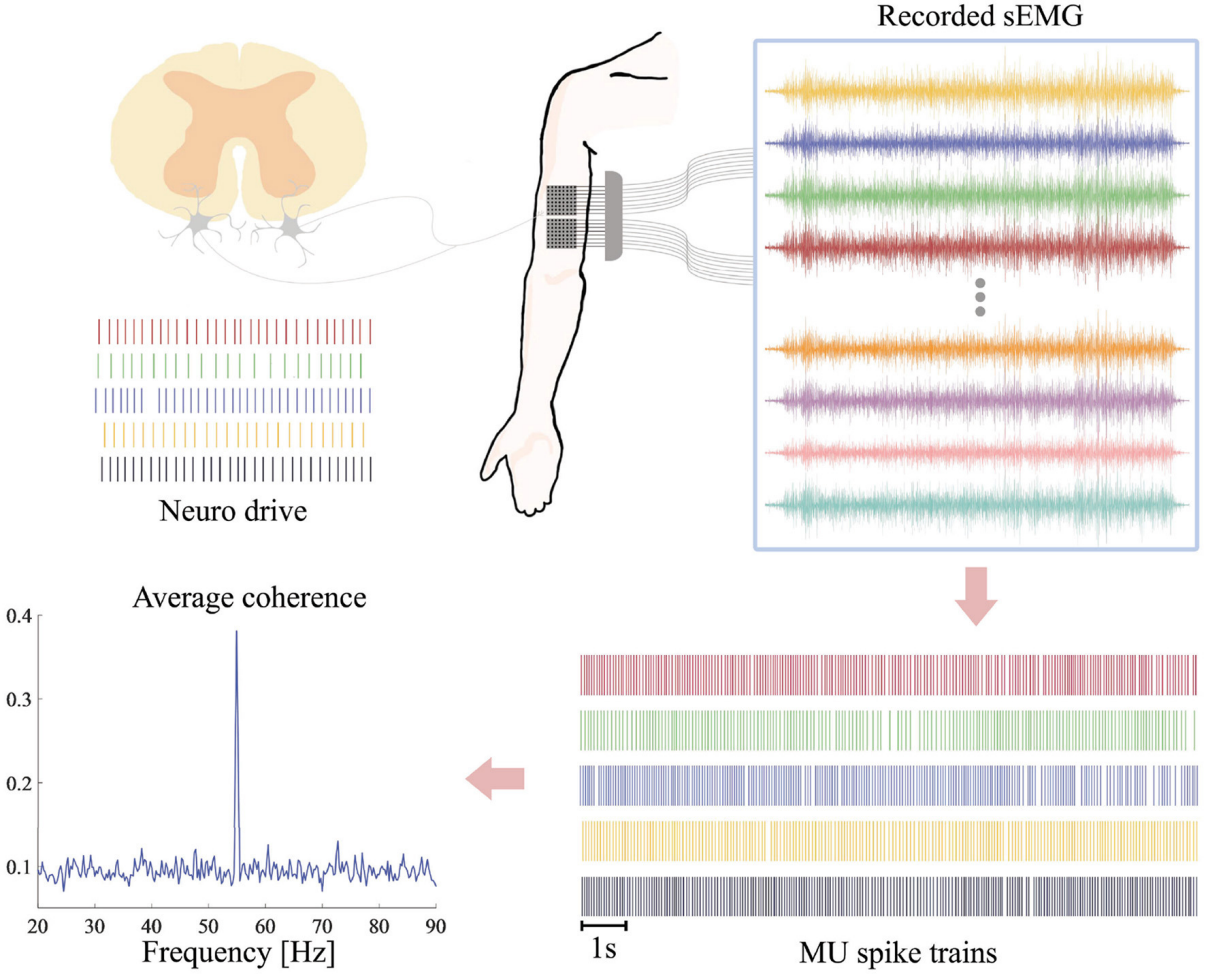
Framework of sEMG processing. Multichannel sEMG are recorded on the biceps brachii during FFS. After preprocessing and notch filtering at the FFS frequency and its first subharmonic, the sEMG signals are decoded into series of MU spike trains. The coherence between different MU spike trains is estimated as a quantitative measure of synchronized activation in different motor neurons.

Surface EMG signals were measured by two 8 × 8 electrode grids (16 rows, eight columns) placed on the biceps brachii of the subject's right arm with the columns along with the direction of the underlying muscle fibers (Figure 2). The diameter of each electrode was 2 mm and the distance between two adjacent electrodes was 8 mm. A circle Ag/AgCl electrode with diameter of 1 cm was placed on the right clavicle of the subject as patient ground. The multi-channel sEMG signals were then amplified by a 128-channel Rafa amplifier (TMS International, Enschede, The Netherlands) and sampled simultaneously at 2,048 Hz with a 24-bit analog-to-digital-conversion resolution.

### 2.3 Measurement protocol

The isometric MVC of each subject was first measured using the adopted experimental setup. The subject sat comfortably on the fitness bench keeping the back straight against the support and the elbow angle at 90 degrees. He/she was then instructed to use his/her maximum effort to keep pulling the handle for 3 s, with the other side of the bar blocked. The force was measured by the load-cell embedded in the bar. The maximum value over the 3 s was considered for one measurement. This procedure was repeated 3 times with a 1-min recovery between two consecutive measurements. The maximum value among the three repeated measurements was considered as the MVC of the subject.

After the MVC measurement, the subject maintained the same position and performed 30-s isometric contractions under different FFS conditions. For all contractions, the baseline force was set to 30% of each subject's MVC. The relatively low baseline level was chosen to ensure reliable sEMG decomposition (Negro et al., 2016). For each contraction, FFS with different amplitudes, i.e., 12.5, 25, and 50% of the baseline, and frequencies, i.e., 20, 30, 40, and 55 Hz, was superimposed on the baseline force, resulting in 12 different FFS trials. Besides, a contraction with the baseline force only was also performed as control condition. Therefore, each subject performed 13 30-s trials in a random order with a recovery time of 2 min in between.

### 2.4 EMG processing and decoding

The 30-s sEMG signals were bandpass filtered between 15 and 450 Hz by a second-order Butterworth filter using the forward and backward filtering method in order to prevent phase shift. A second-order notch filter was applied to remove the power line interference around (± 0.5 Hz) 50 Hz and its harmonics. Another notch filter around (± 0.5 Hz) the FFS frequency and its first harmonic was employed in experiment 1 to eliminate possible motion artifacts generated by vibration-induced movement in the recording electrode grid. After preprocessing, the sEMG signals were decomposed into MU spike trains using the convolutive blind source separation algorithm (Negro et al., 2016; Farina et al., 2017; Jiang et al., 2021).

The basic working principles of the decomposition algorithm are briefly described hereafter. At a discrete time instant *k*, the sEMG signal recorded by electrode *m*, *x*_*m*_[*k*], can be modeled as a convolutive mixture of MU spike trains, expressed as


(1) $$\begin{array}{l} x_{m}\left[k\right]=\overset{N}{\underset{n=1}{\sum }}\overset{L-1}{\underset{l=0}{\sum }}h_{mn}\left[l\right]s_{n}\left[k-l\right]+\omega_{m}\left[k\right],m=1,2,\ldots ,M, \end{array}$$

where *M* is the number of channels (electrodes), *h*_*mn*_[*l*] the action potential of the *n*^*th*^ motor unit recorded at channel *m*, *L* the duration of the action potential, *s*_*n*_[*k*] the spike train (source) of the *n*^*th*^ motor unit, *N* the number of motor unit, and ω_*m*_[*k*] the additive noise at electrode *m*.

A matrix form of Equation (1) that considers all the M channel samples at discrete time instant *k* is given as


(2) $$x[k]=\sum_{l=0}^{L-1}H[l]s[k-l]+\omega [k],$$

where


$$\begin{array}{l} x[k]={\left[\begin{array}{cccc} x_{1}[k] & x_{2}[k] & \ldots & x_{M}[k] \end{array}\right]}^{T},\\ s[k]={\left[\begin{array}{cccc} s_{1}[k] & s_{2}[k] & \ldots & s_{N}[k] \end{array}\right]}^{T},\\ \omega [k]={\left[\begin{array}{cccc} \omega_{1}[k] & \omega_{2}[k] & \ldots & \omega_{M}[k] \end{array}\right]}^{T}, \end{array}$$

and **H**[*l*] is an *M* × *N* matrix.

The aim of the decomposition algorithm is to identify, from the observations **x**[*k*], the largest number of sources, i.e., spike trains **s**[*k*], which are assumed to be either independent or sparse (Farina and Holobar, 2015). To this end, **s**[*k*] is first extended to include the original sources and their *L*−1 delayed versions in order to convert the convolutive mixture in Equation (2) into a linear instantaneous mixture. Accordingly, the observations are also extended by adding *R* delayed versions of each observation in order to maintain a sufficient ratio between the number of observations and that of the sources. The extended model is then given by


(3) $$\tilde{x}[k]=\tilde{H}\tilde{s}[k]+\tilde{\omega }[k],$$

$$\begin{array}{l} \tilde{x}[k]={\left[\begin{array}{cccc} {\tilde{x}}_{1}[k] & {\tilde{x}}_{2}[k] & \ldots & {\tilde{x}}_{M}[k] \end{array}\right]}^{T}\\ \tilde{s}[k]={\left[\begin{array}{cccc} {\tilde{s}}_{1}[k] & {\tilde{s}}_{2}[k] & \ldots & {\tilde{s}}_{N}[k] \end{array}\right]}^{T}\\ \tilde{\omega }[k]={\left[\begin{array}{cccc} {\tilde{\omega }}_{1}[k] & {\tilde{\omega }}_{2}[k] & \ldots & {\tilde{\omega }}_{M}[k] \end{array}\right]}^{T} \end{array}$$

and


(4) $$\tilde{H}=\left[\begin{array}{ccc} {\tilde{h}}_{11} & \cdots & {\tilde{h}}_{1N}\\ \vdots & \ddots & \vdots \\ {\tilde{h}}_{M1} & \cdots & {\tilde{h}}_{MN} \end{array}\right],$$

with


$$\begin{array}{cc} {\tilde{x}}_{m}\left[k\right] & ={\left[\begin{array}{cccc} x_{m}\left[k\right] & x_{m}\left[k-1\right] & \cdots & x_{m}\left[k-R\right] \end{array}\right]}^{T},\;m=1,...,M\\ {\tilde{s}}_{n}\left[k\right] & ={\left[\begin{array}{cccc} s_{n}\left[k\right] & s_{n}\left[k-1\right] & \cdots & s_{n}\left[k-L-R+1\right] \end{array}\right]}^{T},\;n=1,...,N\\ {\tilde{\omega }}_{m}\left[k\right] & ={\left[\begin{array}{cccc} \omega_{m}\left[k\right] & \omega_{m}\left[k-1\right] & ... & \omega_{m}\left[k-R\right] \end{array}\right]}^{T},\;m=1,...,M \end{array}$$

and


(5) $$\begin{array}{l} {\tilde{h}}_{mn}=\left[\begin{array}{cccccc} h_{mn}\left[0\right] & \cdots & h_{mn}\left[L-1\right] & 0 & \cdots & 0\\ 0 & \ddots & \ddots & \ddots & \ddots & \vdots \\ \vdots & \ddots & \ddots & \ddots & \ddots & 0\\ 0 & \cdots & 0 & h_{mn}\left[0\right] & \cdots & h_{mn}\left[L-1\right] \end{array}\right] \end{array}$$

an (*R*+1) × (*L*+*R*) matrix.

Then, the extended sources $\tilde{\text{s}}\left[k\right]$ (discharge timings of individual motor neurons) are estimated by solving Equation (3) using the two-step iteration algorithm proposed in Negro et al. (2016). The first iteration estimates the extended sources by a fixed-point fast ICA algorithm that maximizes the sparseness of the extended sources (Negro et al., 2016; Farina et al., 2017). All the peaks in the estimated source vector (ICA component) are then identified by a peak detection algorithm and then classified into two classes using *K*-means classification. The class with the highest centroid is selected for the estimation of the discharge timings. The second iteration consists of the convolution kernel compensation (CKC) approach aiming at improving the estimation of the source vector (Holobar and Zazula, 2007). It re-calculates each separation vector in ${\tilde{\text{H}}}^{-1}$ from the estimated discharge timings until reaching a minimum variability of discharge, favoring the assumption of regular discharges of the motor neurons.

Besides, as defined in Equation (3), the extracted sources contain the original sources as well as their delayed replicas. In all analysis performed in the present study, the replicas were excluded and only the original sources were considered. Furthermore, by considering a refractory period of 10 ms (Farina et al., 2017), the discharge timings with a interval <10 ms were excluded. Finally, a silhouette measure (SIL), defined as the difference between the within-cluster sums of point-to-centroid distances and the same measure calculated between clusters, was calculated in order to evaluate the quality of the decomposed results (Negro et al., 2016; Dai and Hu, 2019).

### 2.5 Experiment design

Two main experiments are performed in the present study. Experiment 1 presents the effects of FFS on MU activation patterns. Experiment 2 is designed to evaluate the elimination of possible motion artifacts from the decoded MU spike trains.

#### 2.5.1 Experiment 1 (MU discharge pattern during FFS)

All the 18 subjects participated in experiment 1. Each subject performed 13 trials as described in Section 2.3. The decomposition algorithm was applied to the sEMG signal recorded in each trial in order to extract the spike trains (discharge timings) of different MUs. Only spike trains with SIL ≥ 0.9 were considered as reliable MU discharge timings. Any subjects with one or more trials producing less than five reliable MU discharge timings were excluded from further analysis. For each trial of the remaining subjects, the discharge intervals of each MU were calculated from the extracted spike trains. In addition, for each trial, the coherence between each pair of spike trains was calculated in order to assess the degree of synchronized discharge between two motor neurons, as suggested in Dai et al. (2017). Figure 2 presents the schematic diagram of the whole processing steps.

The discrete coherence *C*_*xy*_[*f*] between two spike trains was calculated as the correlation between the two sequences in the frequency domain, as given by


(6) $$\begin{array}{l} C_{xy}\left[f\right]=\frac{|P_{xy}\left[f\right]{|}^{2}}{P_{xx}\left[f\right]P_{yy}\left[f\right]}, \end{array}$$

where *P*_*xy*_[*f*] is the cross-power spectral density between the two spike trains, and *P*_*xx*_[*f*] and *P*_*yy*_[*f*] the auto-power spectral densities of sequence *x* and *y*, respectively. The PSD was calculated using the Welch's approach with a sliding Hanning window of 0.5 s and an overlap of 3/4 window length, in line with previous studies (Dai et al., 2017). Besides, the number of Fourier transform was set to 2 × *f*_*s*_ in order to increase the frequency resolution. The adopted parameters resulted in a 95% confidence limit of 0.027 for the estimated coherence (Dai et al., 2017). The average coherence over all pairs, *C*_*m*_[*f*], was considered for each trial. Furthermore, a synchronization index (SCI) was calculated on *C*_*m*_[*f*] to evaluate the degree of motor neuron synchronization around the FFS frequency *f*_*v*_, given by


(7) $$\mathrm{SCI}=\frac{\sum_{f=f_{v}-2}^{f_{v}+2}C_{m}[f]+\sum_{f=2f_{v}-2}^{2f_{v}+2}C_{m}[f]}{\sum_{f=15}^{120}C_{m}[f]}.$$

Finally, a statistical analysis was performed on the SCIs extracted from the spike trains under different FFS frequencies and amplitudes. Data were normally distributed as suggested by the one-sample Kolmogorov-Smirnov test. As a result, a two-way ANOVA was adopted to access the global effects of FFS frequency and amplitude on the SCI. In addition, a *post-hoc* test with Bonferroni criterion was performed to examine the pairwise difference in FFS frequency and amplitude. The significance level was set to 0.05.

#### 2.5.2 Experiment 2 (validation of motion-artifact removal)

We hypothesized that by notch filtering at the vibration frequency and its first harmonics, on one hand, possible vibration-induced motion artifacts may be eliminated from the decoded discharge timings. On the other hand, such notch filtering will never influence the actual MU discharge timings. With this strategy we could therefore perform reliable interpretation on MU activation patterns under different FFS conditions. Experiment 2 was conducted to validate this hypothesis. To this end, two synthetic datasets were generated based on a real high-density sEMG recorded in experiment 1 without FFS (control condition). This 128-channel sEMG signal was assumed to be motion-artifact free and therefore employed as a baseline signal.

The first synthetic dataset consisted of the selected baseline EMG and a stimulated motion artifacts (a sinusoidal), expressed as


(8) $$\begin{array}{l} S1_{rc}\left[k\right]=x_{rc}\left[k\right]+A1_{rc}\cdot \sin\left[2\pi \frac{f_{v}}{f_{s}}\left(k-\left(r-1\right)\tau_{r}-\left(c-1\right)\tau_{c}\right)\right], \end{array}$$

where *x*_*rc*_ is the baseline EMG with *r* = 1, ⋯, 16 and *c* = 1, ⋯, 8 the indicators of row and column, respectively, *f*_*s*_ = 2, 048 is the sampling frequency, *f*_*v*_ = 20 is the frequency of possible vibration-induced motion artifacts, *A*1_*rc*_ is a scaling factor mimicking motion-artifact amplitude variation in different channels, and τ_*r*_ and τ_*c*_ are time delays of motion artifacts between adjacent rows and columns, respectively. Given the FFS pathway (from the hand to the elbow joint and then the biceps) and the positioning of the recording grids (Figure 2), the simulated motion artifacts were assumed to prorogate through different rows with a velocity of 100 m/s, in line with previous studies (Xu et al., 2015b), resulting in a τ_*r*_≈0.16 sample. Propagation of motion artifacts in different columns of the grid was set to zero. The scaling factor *A*1_*rc*_ is randomly picked between 0.8 and 1.2 of a fixed value that was derived by averaging the amplitude spectrum of all the trials under 20-Hz FFS in experiment 1.

The second synthetic dataset consisted of the same baseline EMG and a simulated EMG with synchronized activation of different motor neurons, as given by


(9) $$\begin{array}{l} S2_{rc}\left[k\right]=x_{rc}\left[k\right]+A2_{rc}\cdot e\left[k-\left(r-1\right)\tau_{r}-\left(c-1\right)\tau_{c}\right]. \end{array}$$

The simulated EMG *e*[*k*] was generated using the convolutive model described in (1), given as


(10) $$\begin{array}{l} e\left[k\right]=\overset{N}{\underset{n=1}{\sum }}\overset{L-1}{\underset{l=0}{\sum }}h_{n}\left[l\right]s_{n}\left[k-l\right], \end{array}$$

where *h*_*n*_[*l*] and *s*_*n*_[*k*] are the action potential and spike train of the *n*^*th*^ motor unit, respectively, *L* the duration of the action potential, and *N* the number of motor units. In the present study, five MUs were considered (*N* = 5) since an exclusion criterion of at least 5 MUs was adopted in experiment 1. Accordingly, for *h*_*n*_[*l*], five action potentials with different morphologies were randomly picked from the decomposition results of experiment 1. In order to mimic synchronized activation, the discharge intervals of different *s*_*n*_[*k*] were assumed to be different but were Gaussian distributed with mean and standard deviation of 50 ± 5 ms, corresponding to a frequency of 20 Hz (the same as the simulated motion artifacts in *S*1). The simulated EMG was assumed to propagate in different rows of the recording grid with a velocity of 4 m/s (Xu et al., 2015a), resulted in a τ_*r*_≈4.1 samples. No time delay was considered between different columns. Besides, the same approach used in *S*1 was adopted to determine the scaling factor *A*2_*rc*_.

**Figure 3:**
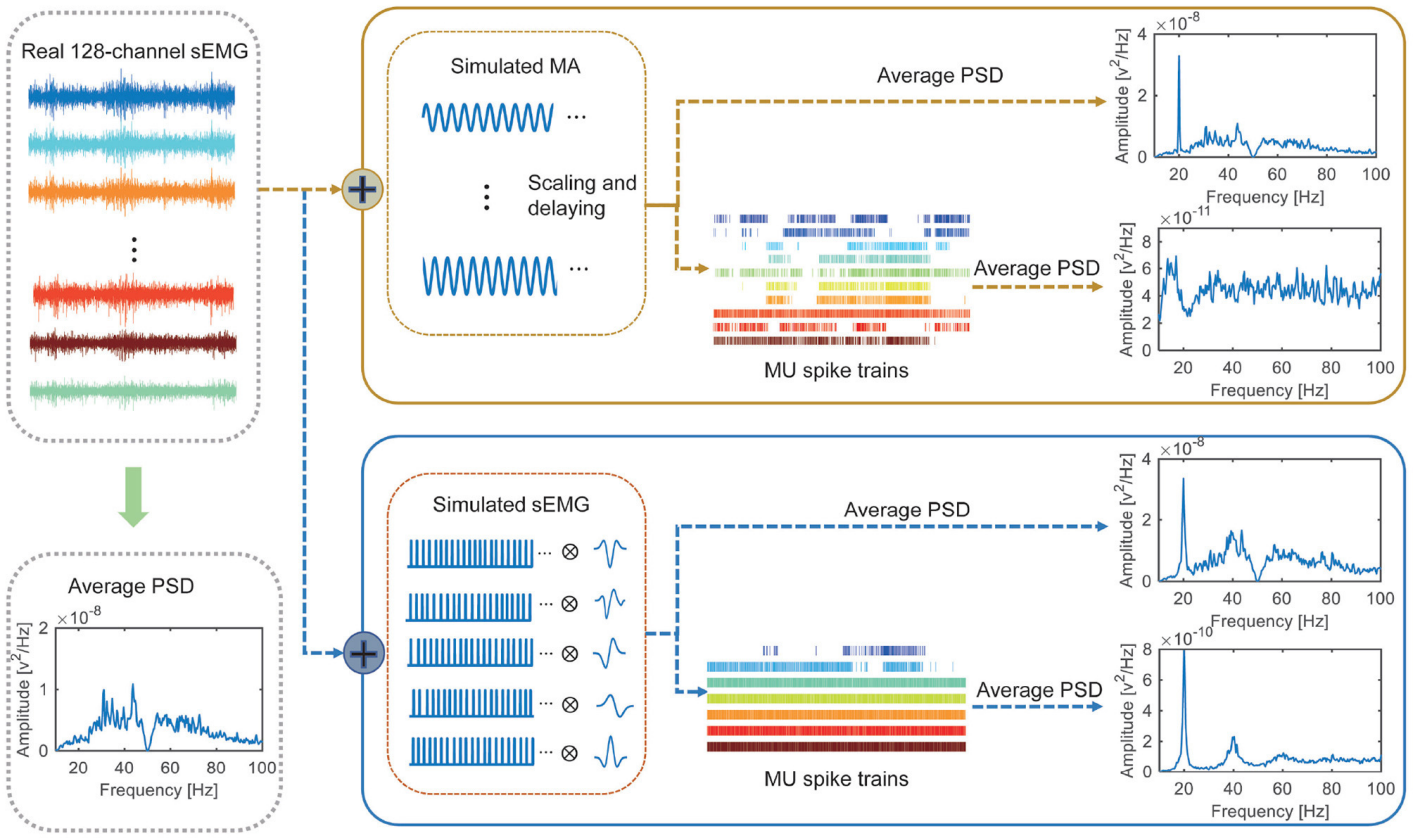
Framework and results of experiment 2. Two synthetic datasets are generated based on a baseline sEMG. For synthetic dataset 1 (consisting of the baseline sEMG and simulated motion artifacts), clear peak presents in the PSD of the original signal but not in the PDS of the decoded MU spike trains. For synthetic dataset 2 (consisting of the baseline sEMG and synchronized activation of 5 MUs), clear peak presents in both the PSD of the original signal and that of the decoded MU spike trains.

Finally, both synthetic datasets were notch filtered around 20 and 40 Hz and then decoded into MU spike trains using the algorithm described in Section 2.4. The PSDs of the spike trains were estimated using the Welch's periodogram and compared. Figure 3 presents the schematic diagram of the whole validation steps.

## 3 Results

### 3.1 Experiment 1 (MU discharge patterns during FFS)

**Figure 4:**
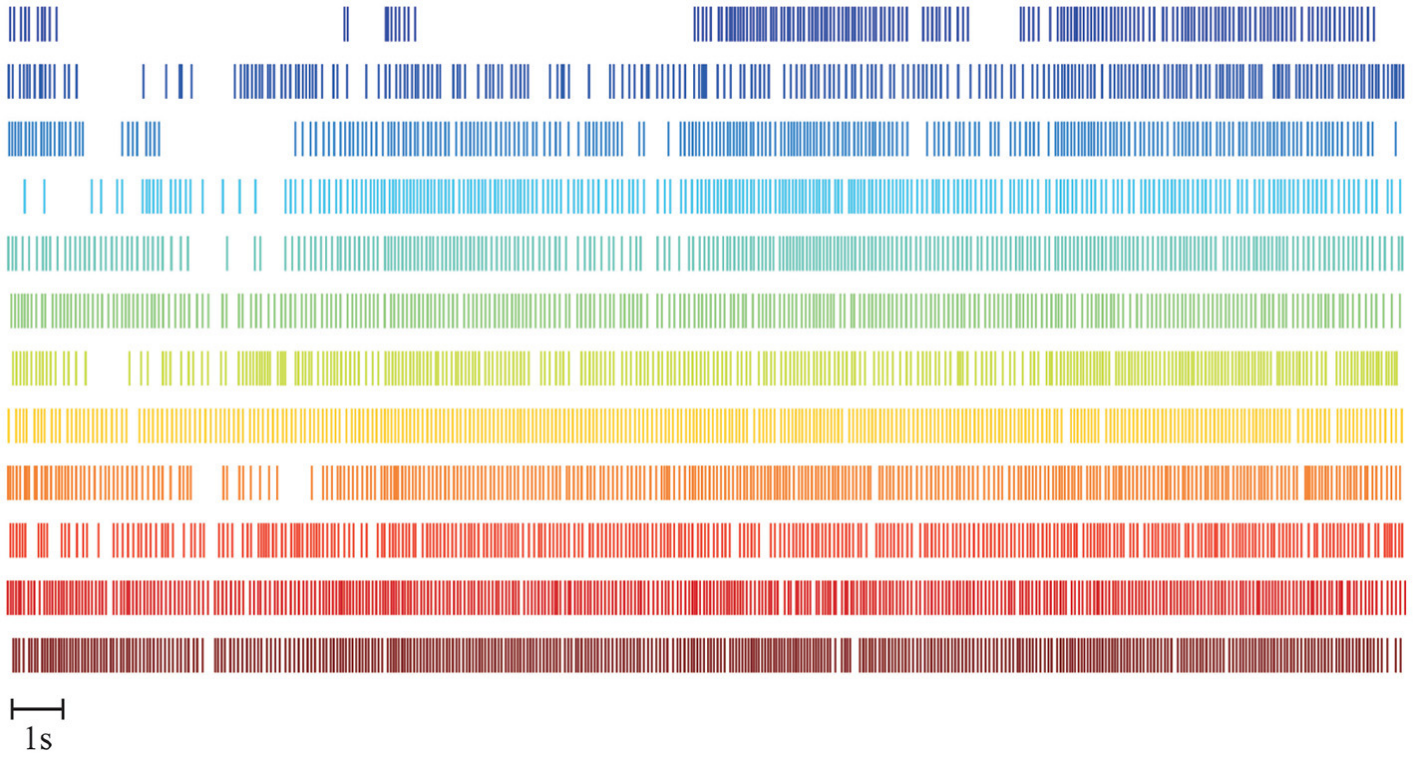
Example of MU spike trains decoded from one FFS trial. Twelve MUs are reliably decoded with an average decoding accuracy of SIL ≈ 0.96.

Four subjects are excluded from this study based on the quality of the decoded MU spike trains, as described in Section 2.5.1. A representative example of the MU spike trains decoded from the sEMG recorded during one FFS trial is shown in Figure 4. In total, 12 MUs have been successfully decoded with an average accuracy, indicated by the silhouette measure (SIL), of ~0.96. For all the trials of the remaining 14 subjects, the number of MUs identified by the decomposition algorithm ranges from 5 to 24. The accuracy of the decomposition (SIL) is between 0.90 and 0.98. The average MU number and SIL for different FFS trials over all subjects are reported in Table 1. No significant difference is observed in the MU number nor the SIL between different FFS conditions.

**Table 1:** Average MU number and SIL for different FFS trials over all subjects.

	**CT^*c*^**	**12.5*****%*** **baseline**				**25*****%*** **baseline**				**50*****%*** **baseline**			
		**20**	**30**	**40**	**55**	**20**	**30**	**40**	**55**	**20**	**30**	**40**	**55 (Hz)**
MUmean^a^	9.5	11.1	10.5	10.2	11.3	10.4	10.1	10.6	10.5	12.0	9.8	10.3	9.9
MUstd	3.3	3.8	3.9	3.4	5.0	4.2	4.5	5.0	4.6	5.0	3.2	4.6	3.5
SILmean^b^	0.96	0.96	0.95	0.96	0.96	0.95	0.95	0.95	0.95	0.95	0.95	0.96	0.96
SILstd	0.01	0.00	0.01	0.01	0.01	0.00	0.01	0.01	0.01	0.00	0.01	0.01	0.01

^a^MUmean and MUstd are mean and standard deviation of the decoded MUs for each trial. ^b^SILmean and SILstd are mean and standard deviation of SIL for each trial. ^c^CT indicates the control condition.

**Figure 5:**
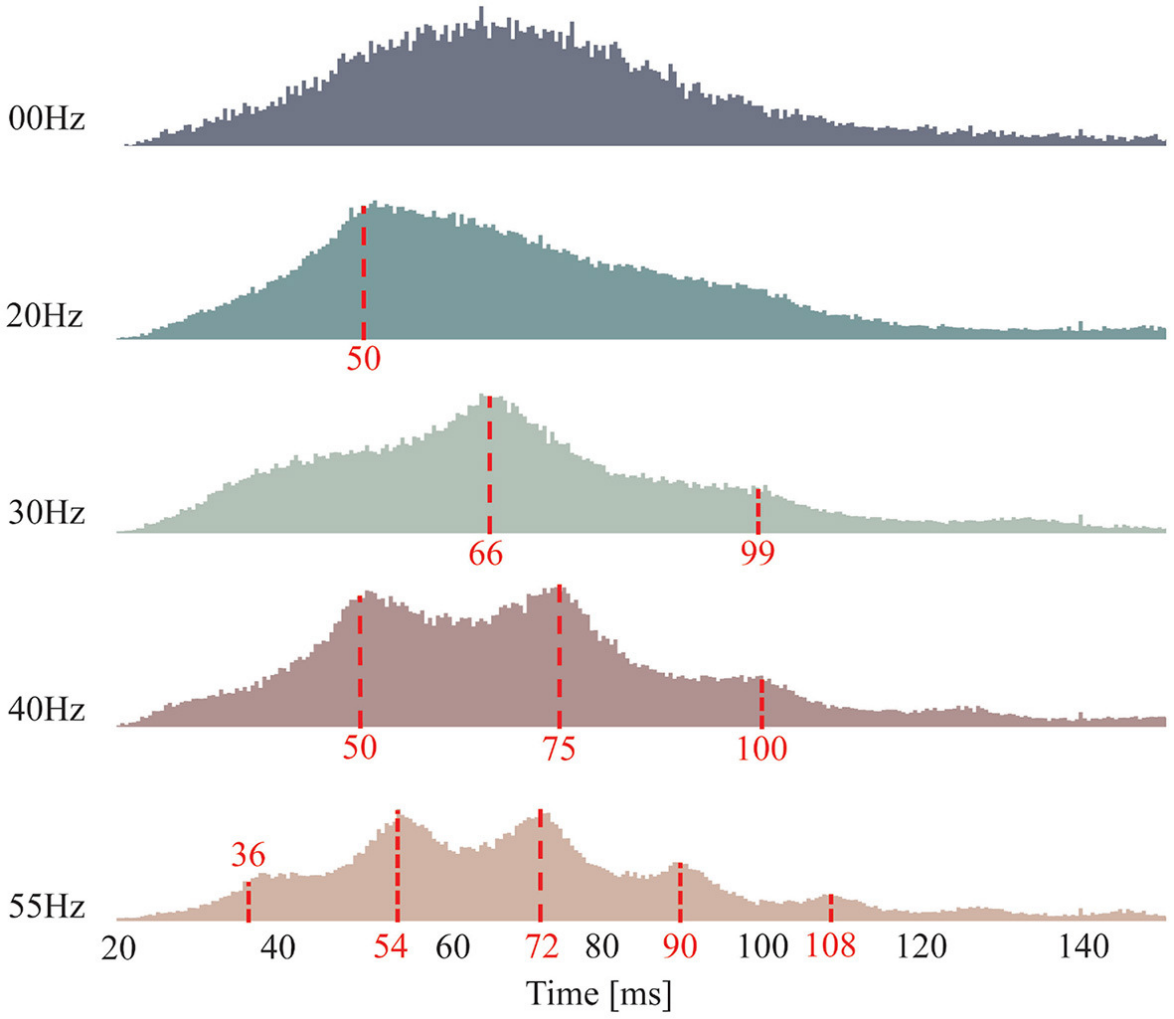
Histogram of MU discharge intervals. Each subfigure consists of the results of the FFS trials of all the subjects with the same frequency but different amplitudes. The control condition determines an approximately Gaussian distribution with mean around 65 ms (corresponds to a firing rate of 15 Hz). Clear peaks present in all FFS trials, indicating significant FFS-induced MU synchronization. Besides, subharmonic synchronization (integer multiples of the duration of the FFS cycle) is observed in the FFS trials with frequency higher than 30 Hz, most probability due to the limited firing rate of the biceps brachii, i.e., maximum 20 Hz on average.

Figure 5 shows the histogram of the discharge intervals for all the FFS trials with the same frequency but different amplitudes, calculated from the decoded MU spike trains over all the remaining 14 subjects. The discharge intervals of the control condition (00 Hz) is approximately Gaussian distributed with a mean around 65 ms and relatively large standard deviations, indicating little or no synchronized MU activation at a specific frequency. In contrast, a clear peak around 50 ms is observed in the histogram of the discharge intervals for 20-Hz FFS trials, implying a synchronized MU activation within the FFS cycle. Furthermore, a dominant discharge interval around 66 ms is observed for the FFS trials at 30 Hz. However, this peak corresponds not to the duration of the FFS cycle but to its integer multiple. This can be attributed to the limited firing rate of the biceps brachii, i.e., maximum average firing rate around 20 Hz, as reported in previous studies (Clamann, 1970; De Luca, 1979). Similarly, synchronized MU activation at the integer multiples of the duration of the FFS cycle is observed for 40- and 55-Hz FFS trials, and the observed integer-multiple synchronization seems to increase with increased FFS frequency.

**Figure 6:**
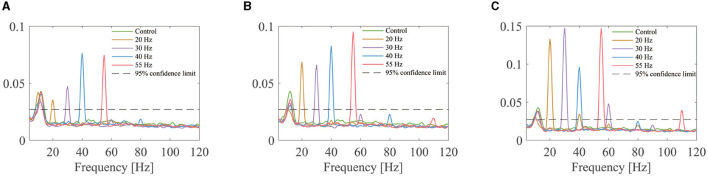
Coherence of the MU spike trains for one subject under different FFS conditions: **(A)** 12.5% baseline force; **(B)** 25% baseline force; **(C)** 50% baseline force. For each trial, the coherence is first calculated between paired MU spike trains, and the average result over all pairs is considered as indicator of MU synchronization. No peak is observed in the control condition, while clear peaks are observed in all FFS trials at the FFS frequency and (or) its harmonics.

An example of the coherence calculated in the decoded MU spike trains of one subject is shown in Figure 6. Sharp peaks are observed at the FFS frequencies and/or their harmonics for all FFS trials irrespective of the amplitude and frequency. However, no predominant peak is observed for the control condition. Given the fact that sEMG is notch filtered around the FFS frequency and its harmonics before decoding, the observed peaks in the coherence demonstrate strongly synchronized discharge in different motor neurons. Similar results have been observed for all the subjects.

**Figure 7:**
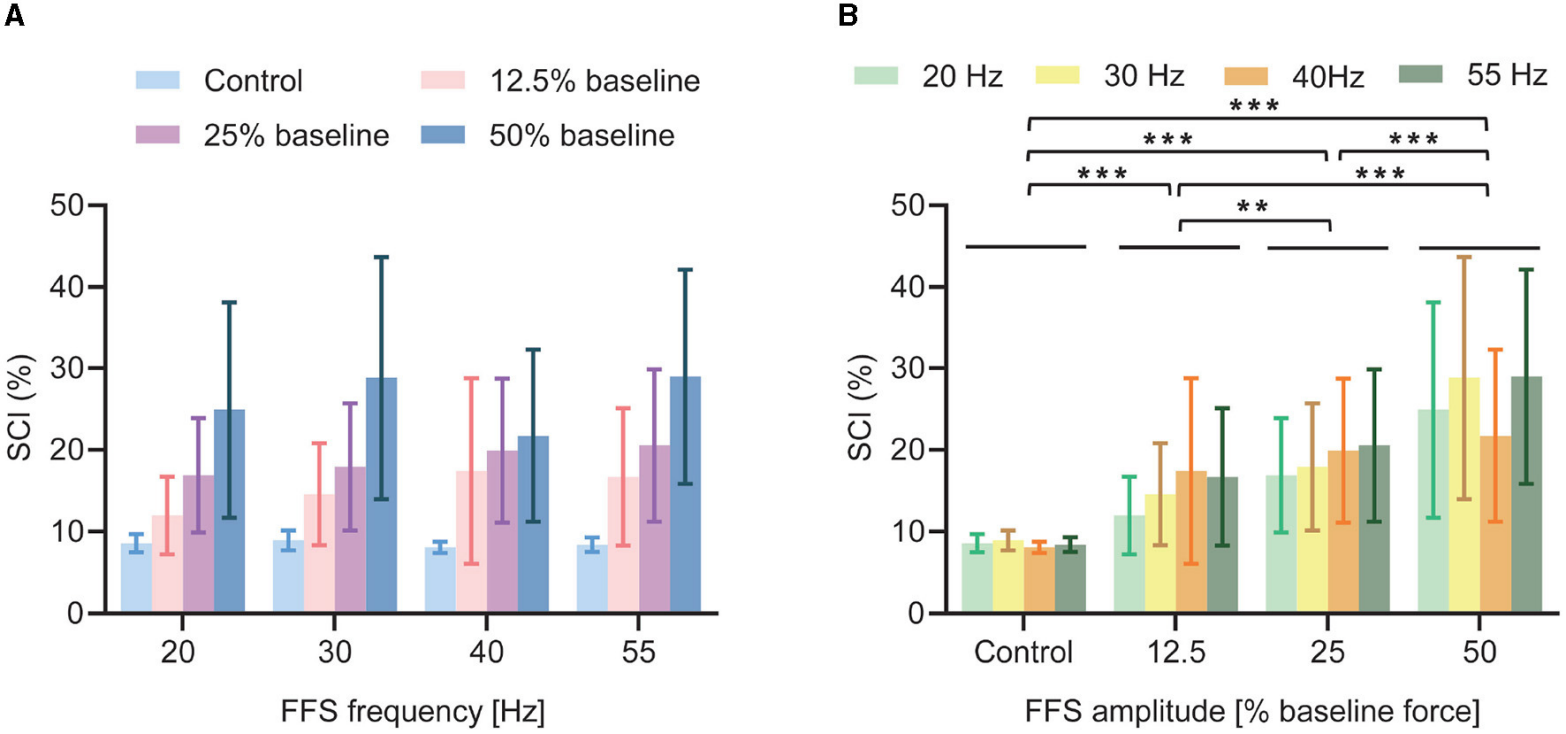
Average SCI (over all subjects) for different FFS trials. All FFS trials determine significantly larger SCI than the control condition: **(A)** No significant effect of FFS frequency is observed for SCI; **(B)** SCI increases significantly with increased FFS amplitude. **p < 0.01; ***p < 0.001.

A synchronization index (SCI) is calculated as a quantitative measure of the synchronized discharge. The average SCI results (over all subjects) for different FFS conditions are shown in Figure 7 and Table 2. All FFS trials produce significantly (*p* < 0.01) higher SCIs than the control condition, confirming FFS-induced MU synchronization. Besides, the SCI increases with increased FFS amplitude. Our two-way ANOVA analysis indicates such an increase to be significant. Similar trend is observed for FFS frequency. However, our statistical analysis reveals no globally significant difference in SCI between different FFS frequencies.

**Table 2:** Average SCI for different FFS trials over all subjects.

	**CT** ^**b**^				**12.5*****%*** **baseline**				**25*****%*** **baseline**				**50*****%*** **baseline**			
	**20**	**30**	**40**	**55**	**20**	**30**	**40**	**55**	**20**	**30**	**40**	**55**	**20**	**30**	**40**	**55 (Hz)**
SCImean^a^	8.57	8.95	8.1	8.42	11.97	14.57	17.43	16.69	16.89	17.92	19.91	20.55	24.93	28.83	21.74	28.99
SCIstd	1.1	1.21	0.69	0.88	4.76	6.23	11.35	8.4	7.01	7.77	8.81	9.31	13.24	14.84	10.52	13.15

^a^SCImean and SCIstd are mean and standard deviation of SCI for each trial. ^b^CT indicates the control condition.

### 3.2 Experiment 2 (validation of motion-artifact removal)

As illustrated in Figure 3, no clear spectral peak can be observed in the power spectral density (PSD) of the adopted baseline sEMG as it is recorded without FFS. A clear spectral peak is presented at 20 Hz in the PSDs of both synthetic datasets, representing the simulated motion artifacts and synchronized MU activation, respectively. After notch filtering and decomposition, no peak appears in the PSD of the MU spike trains decoded from the first synthetic dataset, indicating effective motion-artifact removal by the notch filter. However, a peak at 20 Hz remains in the PSD of the MU spike trains decomposed from the second synthetic dataset after notch filtering. These results indicate that the coherence peaks observed in experiment 1 are caused by synchronized MU activation rather than motion artifacts, confirming the reliability of our results observed in experiment 1.

## 4 Discussion

In the present study, we investigate the effects of a novel vibration exercise modality referred to as FFS on the activation patterns of the motor neurons by dedicated sEMG decomposition. We consider that it is conceptually different from any previous attempt for the analysis of vibration exercise. The main breakthrough is that, instead of using global EMG features that may result in controversial interpretations, we look directly into the discharge timings of the motor neurons decoded from the sEMG. Besides, the spectral components at the FFS frequency and their harmonics are excluded from the decomposition. Our results are therefore straightforward and reliable without any possible bias caused by vibration-induced motion artifacts.

The results reveal significant MU synchronization for all FFS trials, evidenced by the discharge intervals (Figure 5), the peaks in the coherence of the MU spike trains (Figure 6), and the SCIs of different FFS conditions (Figure 7). It is widely accepted that an enhancement in MU synchronization contributes significantly to training-induced increases in muscle strength (Milner-Brown and Lee, 1975; Halliday et al., 1999). In fact, improvement in muscle strength has been extensively reported as a main beneficial effect of vibration training in many previous studies (Burke et al., 1970; Rubin et al., 2001; Cardinale and Wakeling, 2005; Karinkanta et al., 2010). Yet the underlying physiological mechanism has long been unclear. The reported MU synchronization during FFS in the present study may be considered as a primary mechanism contributing directly to the vibration-induced improvement in muscle strength.

Moreover, the observed MU synchronization in the present study seems to be significantly affected by FFS amplitude. For the same baseline force, a higher synchronization is determined with a larger FFS amplitude, as shown in Figure 7. This observation may be ascribed to the spatial and temporal recruitment strategies of the motor neurons. On one hand, according to Henniman's size principle (Henneman et al., 1965), an increase in FFS amplitude may lead to the recruitment of larger and faster motor units, increasing the opportunity for synchronized activation in different motor neurons. Indeed, FFS-induced activation of larger and faster MUs have been reported in previous studies (Xu et al., 2018). On the other hand, an increase in FFS amplitude may also accelerate the firing rate of previously activated MUs, leading to an increase in MU synchronization. FFS may, therefore, produce not only synchronized MU activation but also the activation of more MUs, providing a suitable platform for fitness and rehabilitation programs.

Subharmonic synchronization, i.e., at the integer multiples of the duration of the vibration cycle, is observed for FFS frequencies larger than 30 Hz. Although similar results have been reported in the cat and the human masseter and triceps muscles in previous studies during locally applied MVS (Homma et al., 1972; Godaux and Desmedt, 1975; Burke and Schiller, 1976; Desmedt and Godaux, 1976), the occurrence of MU (subharmonic) synchronization during WBV or FFS has long been doubted (Abercromby et al., 2007; Fratini et al., 2009; Romano et al., 2018; Thompson et al., 2022). The present study confirms, for the first time, the presence of subharmonic MU synchronization during FFS. These observations may be associated with the frequency response of the FFS receptors (mainly the primary spindle endings) as well as the firing rate of the α-motoneuron of the biceps brachii. Although the primary spindle endings are reported responding in 1:1 synchrony up to about 100–150 Hz (Burke et al., 1976; Roll et al., 1989), the average firing rate of the biceps brachii is reported to be ~20 Hz at 100% maximum voluntary contraction (MVC) (Clamann, 1970; De Luca, 1979). In the present study, an average firing rate of around 15 Hz is observed for a sub-maximum (30%) voluntary contraction (Figure 5, control condition), in the range of the 20-Hz limitation. For FFS frequency beyond this limitation, the motor neurons cannot fire with 1:1 synchrony but at integer multiples of the duration of vibration cycle.

The observed subharmonic synchronization in the present study provides a new insight to explain the effects of FFS on muscle training programs. For instance, previous studies have reported that the most effective FFS frequency is around 30 Hz, which has partially been ascribed to possible mechanical resonance of the adopted training system (Mischi and Cardinale, 2009; Xu et al., 2015a). However, we consider the subharmonic synchronization observed in the present study as a more reasonable mechanism to explain this optimal FFS frequency as the mechanical resonance of the system has already been compensated by dedicated system calibration. Since a FFS frequency larger than 30 Hz will lead to synchronized activation of the motor neurons at the integer multiples of the duration of the vibration cycle, it is therefore less effective for training purposes.

Interesting to note that, although the motor neurons synchronize at the subharmonics for FFS frequency higher than 30 Hz, the peaks in the coherence function of the MU spike trains present still at the FFS frequency (and its harmonics) but not the subharmonics, as shown in Figure 6. This is because the coherence is calculated between different motor neurons, which may activate at different, e.g., second or third, subharmonics. Consequently, the peaks in the coherence function locate at the common multiples of different subharmonics. This mechanism may explain our results that the difference in SCI between different FFS frequencies is insignificant, as the SCI is calculated at the FFS frequency and its harmonics, which is not a direct measure of the subharmonic synchronization.

Inspired by the subharmonic synchronization and the location of the coherence peaks, it is reasonable to expect the peaks in the PSD of sEMG to behave in a similar way, as sEMG is the summation of the action potentials of different MUs. Indeed, in the PSD of the sEMG signals recorded under 55-Hz FFS, we do observe sharp peaks at 55 Hz and its harmonics but not the subharmonics, which are in fact the results of subharmonic activation of the motor neurons, as demonstrated in Figure 6. These findings imply that previous studies analysing vibration-induced MU synchronization based on the PSD peaks may unable to identify possible subharmonic activation and therefore overestimate the 1:1 synchrony frequency (100–150 Hz) (Martin and Park, 1997). By decomposing the sEMG signals into MU spike trains, in the present study we can look directly into the discharge intervals of the motor neurons, and therefore provide reliable analysis of the response of the motor neurons to different FFS. This is in fact the main breakthrough of the present study.

Note that a previous study in a small cohort of five subjects has also employed sEMG decomposition for the investigation of the neural drive during FFS (Xu et al., 2019). However, despite the limited subjects, this study lacks the ability to distinguish between real vibration-induce MU synchronization and motion artifacts due to its inclusion of the spectral components at the vibration frequency and its harmonics. And the same for previous PSD-peak based studies, as the nature of these spectral peaks (muscle activity or motion artifacts) has long been debated for decades (Martin and Park, 1997; Abercromby et al., 2007; Fratini et al., 2009; Xu et al., 2015b; Romano et al., 2018). Another breakthrough of the present study lies therefore in motion-artifact removal by notch filtering at the FFS frequency and its harmonics prior to sEMG decomposition. Consequently, possible motion artifacts are completely eliminated from the decoded MU spike trains, permitting the reliability of the present study.

In fact, notch filtering at the FFS frequency and its harmonics cancels also possible muscle activity at those frequencies, whose influence on the decoded MU spike trains is, however, negligible. Intuitively, the discharge timings of each motor neuron are initially extracted by locating the peaks in each ICA component derived from the extended sEMG. Since ICA is a linear transformation, notch filtering on sEMG is equivalent to that on the ICA components. Given the wide spectral range of the ICA components (sEMG), removal of a couple of frequency components may produce little impact on its shape but no influence on the location of the peaks. This interpretation has actually been convinced by our preliminary test.

The original mechanism supporting our approach for motion-artifact removal lies in the different nature of motion artifacts and sEMG, i.e., pure sine waves (Xu et al., 2015b) and convolutive mixture, respectively. Consequently, as demonstrated in our simulation study in experiment 2, motion artifacts are removed from the decoded MU spike trains while synchronized MU activation remains in the PSD of the decoded MU spike trains even with notch filtering prior to the decomposition. These results not only confirm the reliability of our analysis in experiment 1 but also indicate the sEMG PSD peaks observed during FFS to be primarily generated by synchronized MU activation, in line with previous studies (Martin and Park, 1997; Xu et al., 2015b).

The adopted decomposition algorithm is based on blind source separation generally built on the independency of the sources (MU spike trains). However, the extended spike trains in Equation (1) are never independent. Nevertheless, the cost function used in the present study measures also the sparseness of the sources. Yet, some studies argue that synchronization between MU spike trains (particularly evident in the present study) may violate the foundational mathematical assumptions of the blind source separation techniques (Nawab et al., 2008). However, many other studies suggest this idea to not hold for point processes, as the summation of sources with correlated firings is always less sparse than individual sources (Farina and Holobar, 2015; Negro et al., 2016). The linear instantaneous mixture model in Equation (3), derived from the extension of the convolutive mixture model, is therefore assumed to maintain the characteristics required by the proposed blind source separation algorithm (Farina and Holobar, 2015; Negro et al., 2016). Besides, low-quality spike trains (SIL < 0.9) are excluded from the analysis, permitting the accuracy of the decoding and thus the reliability of our analysis on MU discharge patterns during FFS.

Worthily also to note that the current decomposition algorithm can only decompose a small portion of the activated motor units, which is the main limitation of this algorithm. And the discharge interval and the SCI results derived from the decomposed spike trains may in fact be affected by the number of decomposed MUs. However, given the fact that there is no statistically significant difference in MU number across trials (Table 1), our results may represent a general trend of the neuromuscular system in response to different FFS stimulations.

The observed MU (subharmonic) synchronization in the present study may be considered as a manifestation of TVR in FFS. However, some studies have pointed out that the reflex mechanisms responsible for the TVR should be regarded separately from those responsible for vibration-induced timing of MU discharges, as any rhythmical input may synchronize MU discharge to the input (Burke and Schiller, 1976; Hagbarth et al., 1976). Besides, the central nervous system has also been suggested to be involved during vibratory stimulation. It is reported that vibration stimuli applied to the hand or wrist tendons can activate the anterior lobe of the cerebellum as well as many cortex areas, such as the primary somatosensory cortex, the secondary somatosensory cortex, the cortex of the insula, and the supplementary motor area (Fox et al., 1987; Seitz and Roland, 1992; Burton et al., 1993). Unfortunately, based on the results of the present study, it is unable to distinguish the origin of the observed MU synchronization between the spinal reflex and the central nervous system. This may be considered as a main limitation of the present study.

In conclusion, we investigate the effects of FFS on MU discharge patterns by decoding the sEMG signals into MU spike trains using a blind source separation algorithm. Together with notch filtering, we can completely eliminate possible motion artifacts, permitting the reliability of the present study. Our results demonstrate, for the first time, significant MU synchronization of the biceps brachii during FFS. We also find a significant increase in MU synchronization with increased FFS amplitude. Besides, the motor neurons tend to synchronize at integer multiples of the vibration cycle for large FFS frequency (≥30 Hz). Our results reveal the basic physiological mechanism of FFS, and can be employed to explain many debating observations in previous studies involving vibratory stimulation. Therefore, the present study provides a theoretical foundation for introducing FFS into clinical neuromuscular rehabilitation programs. Future studies may focus on discriminating the origin of FFS-induced MU synchronization between spinal reflex and the central nervous systems.

## Data availability statement

The raw data supporting the conclusions of this article will be made available by the authors, without undue reservation.

## Ethics statement

The studies involving humans were approved by Research Ethics Committee of ShanghaiTech University. The studies were conducted in accordance with the local legislation and institutional requirements. The participants provided their written informed consent to participate in this study.

## Author contributions

YX: Data curation, Formal analysis, Investigation, Methodology, Software, Validation, Visualization, Writing – original draft. ZD: Data curation, Formal analysis, Investigation, Methodology, Validation, Visualization, Writing – original draft. AC: Formal analysis, Writing – review & editing. RL: Formal analysis, Writing – review & editing. KW: Data curation, Writing – review & editing. YJ: Data curation, Writing – review & editing. CD: Conceptualization, Supervision, Methodology, Writing – review & editing. LX: Funding acquisition, Conceptualization, Project administration, Resources, Supervision, Writing – review & editing.
